# Development of metachronous rectal cancers in a young man with dyskeratosis congenita: a case report

**DOI:** 10.1186/s13256-019-2044-5

**Published:** 2019-04-27

**Authors:** Motoko Watanabe, Gou Yamamoto, Kenji Fujiyoshi, Yoshito Akagi, Miho Kakuta, Yoji Nishimura, Kiwamu Akagi

**Affiliations:** 1 Department of Molecular Diagnosis and Cancer Prevention, Saitama Cancer Center, 780 Komuro, Ina-machi, Kitaadachi-gun, Saitama, 362-0806 Japan; 2 Department of Surgery, Kurume University, Fukuoka, Japan; 3 Division of Gastroenterological Surgery, Saitama Cancer Center, Saitama, Japan

**Keywords:** Dyskeratosis congenita, Rectal cancer, *DKC1*, Missense variant, X-linked recessive

## Abstract

**Background:**

*DKC1* (dyskerin pseudouridine synthase 1) is a causative gene for X-linked dyskeratosis congenita. Approximately 8% of patients with dyskeratosis congenita have malignancy, but information about the development of malignancy in patients with dyskeratosis congenita is limited.

**Case presentation:**

A young Japanese patient with bone marrow failure developed metachronous rectal adenocarcinomas at the ages of 16 and 18 years. He had no family history of cancer. Microsatellite instability testing with rectal tumor tissue demonstrated low-level microsatellite instability. To clarify whether any cancer susceptibility genes were involved in the development of rectal cancer, RNA sequencing was performed. Cancer-related genes were assessed, and a c.361A>G (p.Ser121Gly) germline variant was detected in *DKC1*. The same missense variant was previously reported in two patients with dyskeratosis congenita as a pathogenic variant, but those patients did not develop malignancies.

**Conclusions:**

Our patient developed rectal cancer at an early age of onset compared with the previously reported typical onset age of patients with dyskeratosis congenita. *DKC1* might be involved in predisposition to colorectal cancer in young adulthood; therefore, appropriate surveillance may be considered.

## Background

Dyskeratosis congenita (DC), which is also known as Zinsser-Cole-Engman syndrome, was originally reported by Zinsser in 1906 [1]. It is a rare inherited bone marrow failure syndrome and is classically characterized by dysplastic nails, lacy reticular pigmentation of the upper chest and/or neck, and oral leukoplakia. At least two of these features or one feature plus two or more of the following findings are suspicious for DC: eye abnormalities (epiphora, blepharitis, sparse eyelashes, ectropion, entropion, trichiasis), dental abnormalities (caries, periodontal disease, taurodontism), alopecia, developmental delay, short stature, microcephaly, hypogonadism, esophageal stenosis, urethral stenosis, liver disease, osteoporosis, and avascular necrosis of the hips or shoulders [2]. In addition, individuals with DC often develop pulmonary fibrosis and malignancies. However, the phenotype is highly variable. The main causes of mortality are bone marrow failure and immune defects (60–70%), followed by pulmonary complications and cancers [3–5]. Affected patients have very short telomeres [6, 7] and/or pathogenic variants in one of the telomere biology genes: *ACD*, *CTC1*, *DKC1*, *NHP2*, *NOP10*, *PARN*, *RTEL1*, *TERC*, *TERT*, *TINF2*, and *WRAP53*. However, the mode of inheritance of DC varies by genes. *TERC* and *TINF2* demonstrate an autosomal dominant (AD) pattern, and *CTC1*, *NHP2*, *NOP10*, *PARN*, and *WRAP53* have an autosomal recessive (AR) pattern. *RTEL1*, *TERT*, and *ACD* show either an AD or AR pattern.

*DKC1* is a causative gene for X-linked recessive inheritance of DC [OMIM:MIM305000] [8–10]. In this case, in general, only males are affected by DC. *DKC1* codes for dyskerin, which functions in both the stabilization of the telomerase RNA component and the pseudouridylation of ribosomal RNA molecules [6, 11, 12].

Approximately 8–10% of patients with DC develop malignancies [3, 13]. The most frequent tumor in patients with DC is head and neck squamous cell carcinoma (40%), followed by skin and anorectal cancers. The incidence of malignancy is 11-fold higher in patients with DC than in the general population.

In this report, we describe a patient with a *DKC1* missense variant, c.361A>G (p.Ser121Gly), who developed multiple rectal cancers in young adulthood after bone marrow failure. Because the clinical features of this patient with DC were atypical except for bone marrow failure, it was difficult to make an accurate diagnosis. Comprehensive RNA sequencing enabled us to diagnose the patient as having DC.

## Case presentation

A 15-year-old Japanese boy was admitted to our hospital because of bloody stool. He had chronic otitis media at the age of approximately 2 years and purpuric lesions on his face and feet at the age of 9 years. At the age of 12 years, he had erythrocytopenia and thrombocytopenia (total white blood cells, 3.1 × 10^3^/μl; red blood cells, 2.63 × 10^6^/μl; hemoglobin, 9 .6 mg/dl; platelets, 7 × 10^3^/μl) and was diagnosed with aplastic anemia.

Since progression of his aplastic anemia, treatment with steroid had been performed after high-dose gamma-globulin therapy at the age of 14 years at another hospital.

**Fig. 1:**
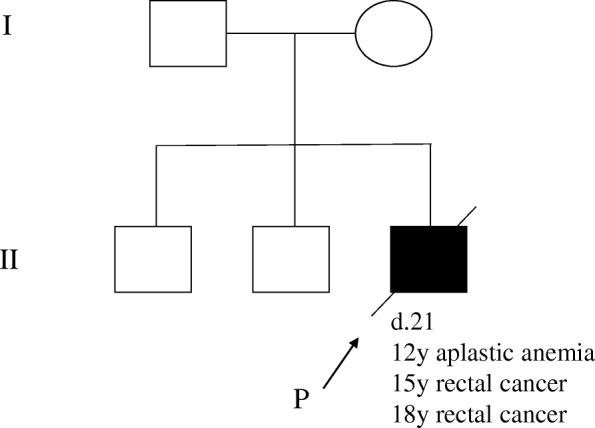
Family pedigree. No other family members were affected

He had no smoking or drinking habit. His two brothers and parents had no symptoms. His parents are not consanguineous (Fig. 1). On examination, his temperature was 36.4 °C, pulse 72 beats/min, blood pressure 132/64 mmHg, respiratory rate 20 breaths/min, weight 47 kg, and height 165 cm. He had no developmental disorders or intellectual disability. The results of his physical and neurological examinations were normal. His laboratory findings were as follows: hemoglobin 8.7 g/dl; hematocrit 26.1%; white blood cell count 1660/mm^3^ with 53% neutrophils, 32% lymphocytes, 13% monocytes, and 2% eosinophils; platelet count 47,000/mm^3^; red blood cell count 2,860,000/mm^3^; sodium 138 mmol/L; potassium 3.5 mmol/L; chloride 104 mmol/L; urea nitrogen 10 mg/dl; creatinine 0.54 mg/dl; glucose 95 mg/dl; total protein 7.2 g/dl; albumin 3.8 g/dl; alanine aminotransferase 30 U/L; aspartate aminotransferase 34 U/L; alkaline phosphatase 561 U/L; total bilirubin 0.8 mg/dl; and C-reactive protein < 0.3 mg/dl. His urinalysis values were as follows: specific gravity 1.020, protein negative, and glucose negative. Test results for antibodies to hepatitis B virus surface antigen, hepatitis C virus antibodies, human immunodeficiency virus antibodies, and *Treponema pallidum* antibodies were negative. Karyotype examination of peripheral blood was normal (46,XY).

**Fig. 2:**
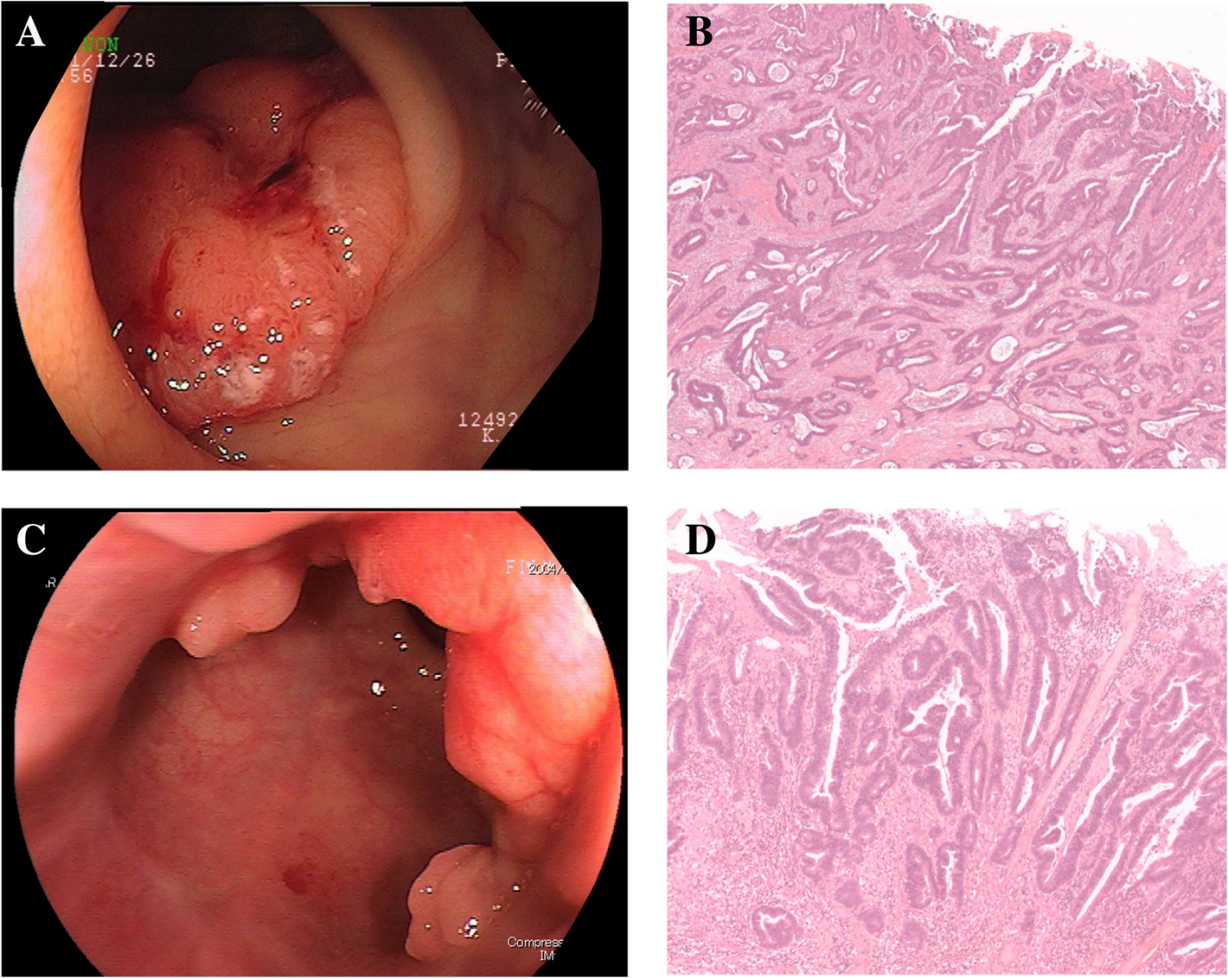
**a** Endoscopic examination showed an ulcerative tumor in the rectum. **b** Pathological examination of the resected first rectal tumor revealed well-differentiated or moderately differentiated adenocarcinoma. **c** Endoscopic examination showed superficial elevated tumor in the rectum. **d** Pathological examination of the resected second rectal tumor revealed well-differentiated or moderately differentiated adenocarcinoma

**Fig. 3:**
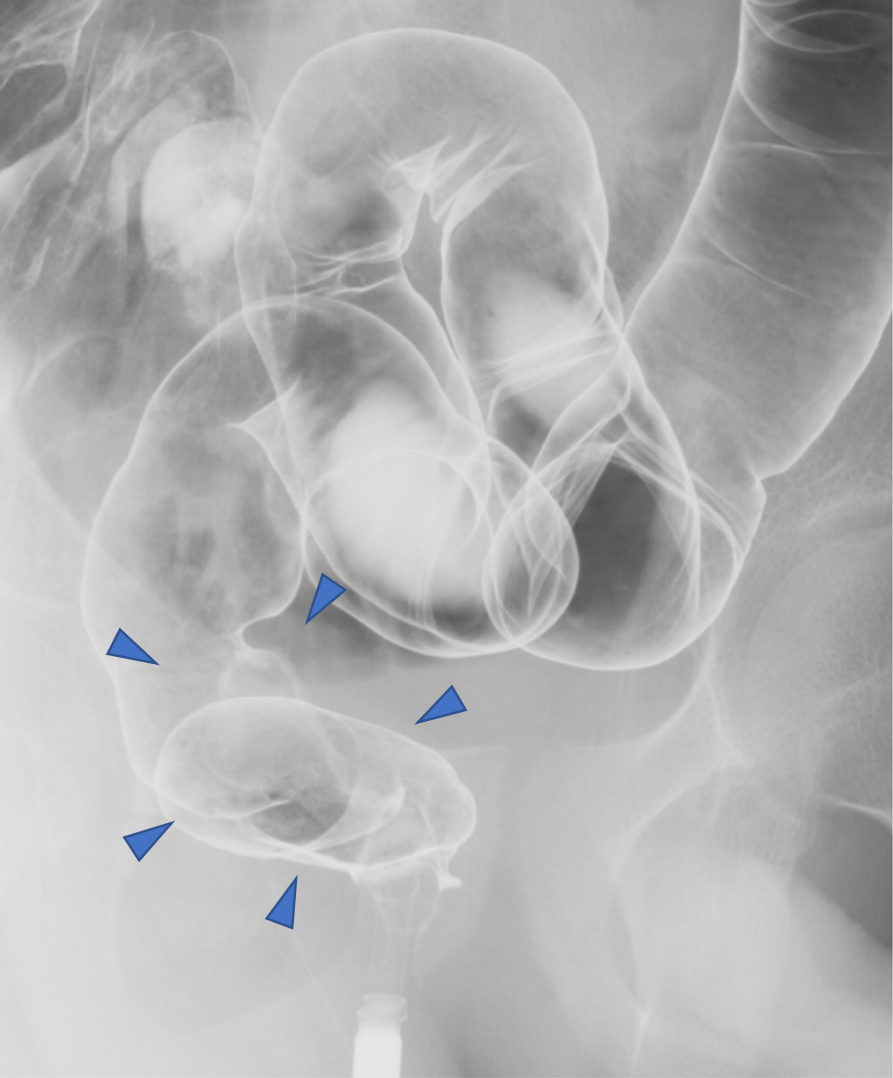
Barium enema showed excavated lesion with raised margins (*arrowheads*) in the rectum

**Fig. 4:**
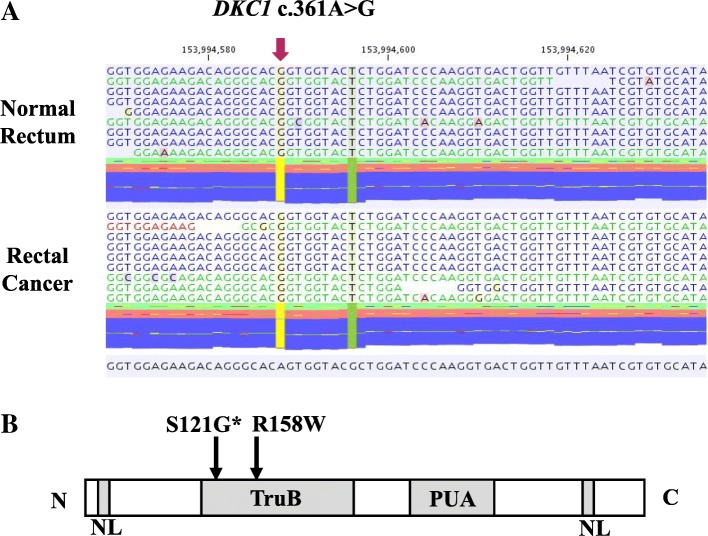
**a** Sequencing analysis of normal and rectal cancer tissues. The *red arrow* indicates the position of the missense mutation c.361A>G. **b** The dyskerin protein includes the nuclear localization signals (NL) and the TruB and PUA (pseudouridine synthase and archaeosine transglycosylase) domains. Previously reported missense mutations in the TruB domain are shown. The mutation identified in the present case was p.Ser121Gly and is indicated by the *asterisk*

Endoscopic examination showed an ulcerative tumor in the rectum (Fig. 2a), and barium enema demonstrated an excavated lesion with raised margins in the lower rectum (Fig. 3, arrowhead). Lower anterior resection with left lateral lymph node dissection were performed for rectal cancer. The tumor was a protruding 2.8-cm × 2.5-cm mass in the rectum with well or moderate differentiation (Fig. 2b) and full-thickness infiltration (pT4N1M0, stage IIIB). Neither *KRAS* nor *BRAF* mutation was detected in the rectal cancer. Dilation of the splenorenal shunt vein (7 mm), moderate splenomegaly, an accessary spleen, dilation of the portal vein system, and hepatomegaly were also observed. The patient had neither chronic hepatitis nor hepatic cirrhosis. Oral leukoplakia was observed when he was 17 years old. He was admitted to this hospital again because of bloody stool at the age of 18 years. On examination, his temperature was 36.8 °C, pulse 74 beats/min, blood pressure 102/56 mmHg, respiratory rate 23 breaths/min, weight 43,8 kg, and height 168 cm. The results of his physical and neurological examinations were normal. Endoscopic examination showed a superficial elevated tumor in the rectum (Fig. 2c). Metachronous rectal cancer was resected through transanal partial proctectomy. The tumor was a protruding 3.5-cm × 1.5-cm mass in the rectum with well or moderate differentiation (Fig. 2d) and submucosal infiltration (pT1N0M0, stage I). One year later, the rectal cancer recurred, and persistent anal bleeding and progressive pancytopenia were observed. The patient underwent bone marrow transplant at the age of 20 years. However, he died of progressive hepatic failure at the age of 21 years. Autopsy has not been performed. Because this patient developed juvenile-onset multiple rectal cancers and hematological malignancy, we suspected constitutive mismatch repair deficiency (CMMRD) syndrome, which is a childhood cancer predisposition syndrome especially including brain tumor, colorectal tumor, and hematological malignancies involving biallelic germline pathogenic variants of mismatch repair genes. However, microsatellite instability (MSI) testing with tumor tissue demonstrated low-level MSI, indicating that the possibility of CMMRD was low. In order to pursue further causes, whole-transcriptome analysis of frozen rectal cancer samples of the patient was conducted to elucidate the characteristics of the tumors, and the missense variant c.361A>G (p.Ser121Gly) in the *DKC1* gene on chromosome X was detected (Fig. 4a). It was confirmed as a germline hemizygous variant in normal tissue.

## Discussion and conclusions

Because this patient did not have typical clinical features and family history of DC except for bone marrow failure, the diagnosis of this case was made by RNA sequencing. The patient developed metachronous rectal cancers after bone marrow failure at an early onset age compared with previously reported patients with DC. Genomic DNA analysis confirmed a germline missense variant, c.361A>G (p.Ser121Gly), in *DKC1*. This variant is located in exon 5 of *DKC1* and resides in the TruB domain, pseudouridine synthase motif (Fig. 4b). The c.361A>G (p.Ser121Gly) variant in *DKC1* was previously reported as a causative variant for Hoyeraal-Hreidarsson (HH) syndrome, which is a severe type of DC and shows additional features, including intellectual disability and cerebellar hypoplasia [14, 15]. However, our patient had neither intellectual disability nor cerebellar hypoplasia, suggesting that there is marked phenotypic variety among patients with the same variants (Table 1). Another missense variant in the TruB domain reported to date was p.Arg158Trp, and it also led to HH syndrome [16, 17]. The variants in the TruB domain have been hypothesized to be associated with a severe phenotype due to greater defects in the pseudouridylation activity of dyskerin than those of variants lying outside the TruB domain [3]. The severity of the clinical phenotype in patients with DC has been suggested to correlate with telomere length, but we were not able to examine telomere length in our patient [17].

**Table 1:** Characteristics of patients with Ser121Gly substitution in *DKC1*

	Case 1	Case 2	Present case
Sex	M	M	M
Growth failure	+	+	–
Microcephaly	+	+	–
Intellectual disability	+	+	–
Cerebellar hypoplasia	+	+	–
AA, age at onset (years)	1	1.5	12
Recurrent infections	+	+	+
Cancer	n.d.	n.d.	Rectal cancer
Age at death (years)	5	n.d.	21
Reference	Knight *et al*., 1999 [9]	Knight *et al*., 1999 [14]	

*Abbreviations: M* Male, *AA* Aplastic anemia, *n.d.* Not described

According to a previous report on colorectal cancer, the average age at diagnosis of rectal cancer in six patients with DC was 28 years, whereas two patients with DC with colon cancer were diagnosed at 20 and 25 years old, respectively [13]. One patient with DC with rectal cancer has been reported from Japan: a 24-year-old Japanese man with DC complicated by noncirrhotic portal hypertension, signet ring carcinoma of the rectum, and *Pneumocystis carinii* pneumonia [18]. Our patient with DC also was complicated by noncirrhotic portal hypertension and rectal cancer, but the tumor demonstrated well-differentiated or moderately differentiated adenocarcinoma. Thus, patients with DC develop colorectal cancers earlier than the general population does. It is suggested that defects in pseudouridylation could lead to an impairment of translation, probably including tumor suppressors [19, 20]. Because the previously reported patients with variants in the TruB domain had a severe phenotype and poor prognosis, they probably died before cancer developed. Proteomic analysis may elucidate whether the impairment of translation due to defects in the pseudouridylation activity of dyskerin is related to the development of malignancy in patients with DC. Although it is unknown how *DKC1* is involved in cancer development, DC often develops various tumors in the digestive system, such as in the rectum, stomach, esophagus, colon, pancreas, and liver, at an early age.

In conclusion, our patient developed rectal cancers twice at an early-onset age compared with other previously reported patients with DC who developed colorectal cancer. *DKC1* might be involved in predisposition to colorectal cancer in young adulthood; therefore, appropriate surveillance may be required, such as with fecal occult blood test, digital rectal examination, and/or endoscopic examination from the age of 10 years, considering our patient’s case.

## Abbreviations

AD: Autosomal dominant
AR: Autosomal recessive
CMMRD: Constitutive mismatch repair deficiency
DC: Dyskeratosis congenita
HH: Hoyeraal-Hreidarsson
MSI: Microsatellite instability
